# Genome sizes of 227 accessions of *Gagea* (Liliaceae) discriminate between the species from the Netherlands and reveal new ploidies in Gagea

**DOI:** 10.1186/s40064-015-1167-4

**Published:** 2015-08-05

**Authors:** B J M Zonneveld, B te Linde, L-J van den Berg

**Affiliations:** NBC Naturalis, Herbarium Section, P.O. Box 9517, 2300 RA Leiden, The Netherlands; Berglinde BV, Dorpstraat 50, 6909 AL Babberich, The Netherlands

**Keywords:** *Gagea*, The Netherlands, DNA 2C-value, Genome size, Ploidy level, New decaploid *G. pratensis*

## Abstract

Nuclear genome size, as measured by flow cytometry with propidium iodide, was used to investigate the relationships within the genus *Gagea* (Liliaceae), mainly from the Netherlands. The basic chromosome number for *Gagea* is x = 12. The inferred ploidy in the Dutch and German accessions varies from diploid to decaploid. Consequently there is a large range of genome sizes (DNA 2C-values) from 14.9 to 75.1 pg. Genome sizes are evaluated here in combination with the results of morphological observations. Five species and the hybrid *G.* × *megapolitana* are reported. Apart from 14 diploid *G. villosa*, six plants of *G. villosa* with an inferred tetraploidy were found. For the 186 Dutch accessions investigated 85 turned out to be the largely sterile *G. pratensis* (inferred to be pentaploid). Inferred tetraploid and hexaploid *G. pratensis* were found in 30 and 20 localities, respectively. In one locality an inferred decaploid (10×) plant was found that could represent a doubled pentaploid *G. pratensis*. An inferred decaploid *G. pratensis* was never reported before. The genome size of *Gagea* × *megapolitana* from Germany fitted with its origin as a cross between the two hexaploids *G. pratensis* and *G. lutea*. *Gagea spathacea* from the Netherlands was inferred to be nonaploid as was recorded from plants across Europe. The aim of the study was to use flow cytometry as a tool to elucidate the taxonomic position of the Dutch *Gagea*.

## Background

The genus *Gagea* Salisb. conmprizes about 275 species. In the World Checklist for *Gagea* (Govaerts 2006) 594 names were listed. It is a genus of small bulbous plants in the family Liliaceae, endemic to Eurasia and North Africa. A single circumpolar species, a former *Lloydia* is now included in *Gagea* (Peruzzi 2012). The greatest number of species can be found in Kazakhstan in the Tien Shan and Pamir-Alai. This coincides with the greatest richness of *Tulipa* (Zonneveld 2010). In Flora Neerlandica (van Oostrom and Reichgelt 1964) four species are recorded for The Netherlands and in Heukels Flora of The Netherlands (van der Meijden 2005) a fifth is added.

To elucidate the relationships between *Gagea* species, the classical taxonomic traits based on morphological characters, chromosome numbers (Peruzzi 2003, 2012) and sequencing data (Peterson et al. 2008; Zarrei et al. 2009) are here supplemented with data on nuclear DNA content. From only five species genome size was determined earlier (Greilhuber et al. 2000; Vesely et al. 2011; Leitch et al. 2007). Taxonomy of *Gagea* is rather difficult and the main useful characters so far are: the chromosome numbers, the number and type of bulbils, the number and width of the leaves, the presence of red coloration at the base of the leaf, the hairiness of the flower stalk, the shape of the petals and the number of flowers on a scape. Newer investigations are also based on morpho-anatomical data (Peruzzi 2012).

**Fig. 1:**
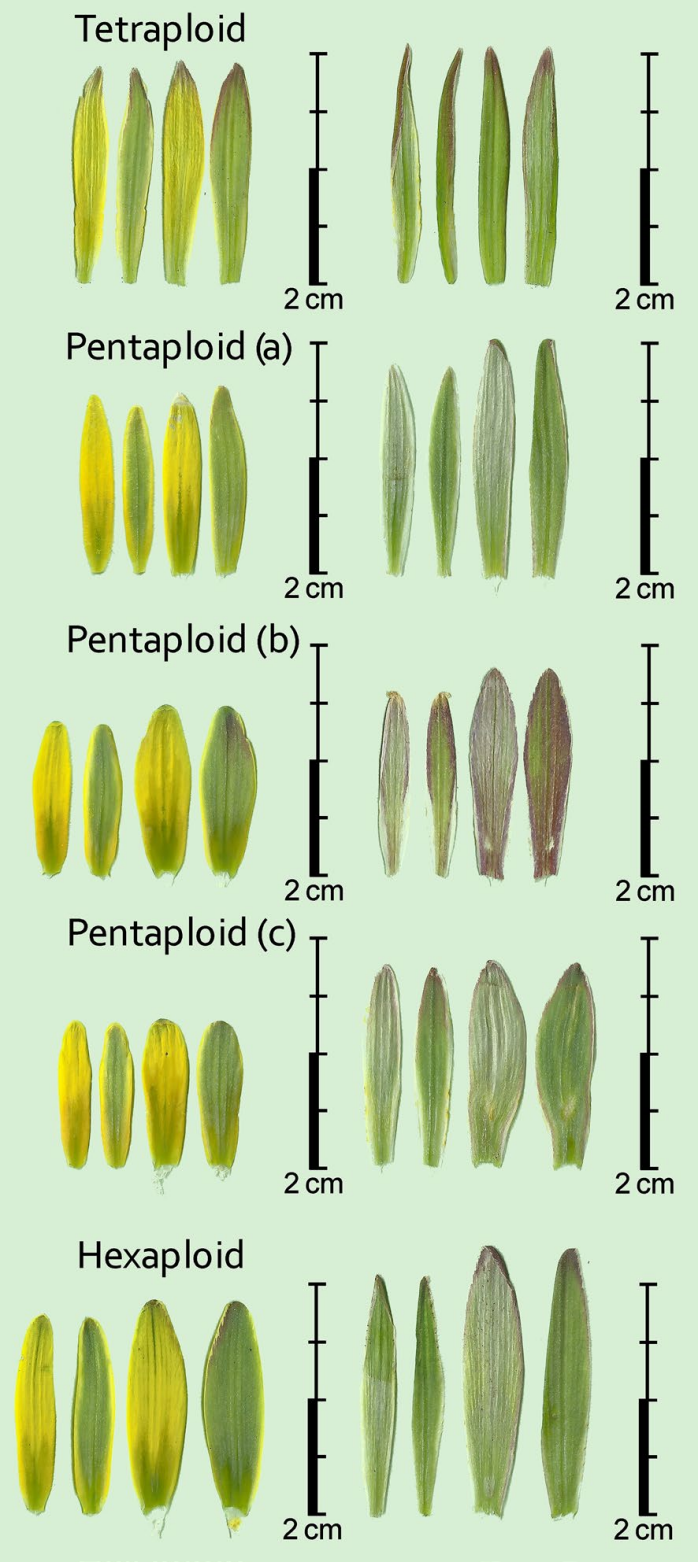
Scans of petals of *Gagea pratensis* in the Netherlands. In the *left column* petals from a fresh inflorescence. On the *left* the inner petals (*left* the *upper side* and on the *right* the *bottom side*). On the *right* the outer petals (*left* the *upper side* and on the *right* the *bottom side*). In the *right column* petals from an inflorescence after bloom. On the *left* the inner petals (*left* the *upper side* and on the *right* the *bottom side*). On the *right* the outer petals (*left* the *upper side* and on the *right* the *bottom side*). The tetraploid gagea is collected in a park near the river Berkel in Almen. The pentaploid (a) gagea is collected in a churchyard in Wassenaar. (All gageas in the western part of the Netherlands are of this type). The pentaploid (b) is collected in a road verge near Fromberg. The pentaploid (c) is collected in a park in Zutphen. (These large gageas resemble *Gagea megapolitana*). The hexaploid gagea is collected in a park near the river IJssel in Deventer.

186 different accessions from The Netherlands were measured in an attempt to understand the relationships within the Dutch gageas. These values were compared with an additional 41 taxa from Germany. Nuclear DNA content can conveniently be measured by flow cytometry using propidium iodide, a stoichiometric DNA stain that intercalates in the double helix. Where many species in a genus have the same chromosome number, differences in DNA 2C-value have proven to be very effective in delimiting infrageneric divisions in a number of taxa (Ohri 1998). The evolution of genome size (Greilhuber 1979) has received increased attention during recent years (Greilhuber 2005). The smallest angiosperm genome size reported so far is for *Genlisia margarethae* Hutch. with 2C = 0.13 pg (Greilhuber et al. 2006). The record holders for maximum genome size were for eudicots *Viscum album* L. with 2C = 205.8 pg and for monocots *Paris japonica* with 2C = 304.5 pg (Pellicer et al. 2010). Flow cytometry was successfully used to measure the 2C-value for the genera *Hosta* Tratt., *Helleborus* L., *Clivia* Lindl., *Nerine* Herb., *Agapanthus* L’Hér., *Galanthus* L., *Narcissus* L., *Gasteria* Duval*. Tulipa* L. etc. by Zonneveld (2001, 2003, 2008, 2009, 2010), Zonneveld and Van Iren (2001), Zonneveld and Duncan (2003, 2006), and Zonneveld et al. (2003, 2012). In this paper it is shown that genome size is helpful to discriminate between the species of *Gagea* from The Netherlands (Fig. 1).

Nuclear DNA content as measured by using flow cytometry may conveniently be used to produce systematic data. It is applicable even in dormant bulbs or sterile plants for the monitoring of the trade in bulbous species. In the case of *Gagea*, it is difficult to ascribe a plant to a taxon in the often non-flowering state. Genome size is a good way to determine the species and their ploidy. A different genome size infers usually a different ploidy or a different species. However, the reverse is not true: if plants have the same genome size it does not automatically mean that they are the same species, it might be a coincidence.

Based on van den Berg and te Linde (2003) and new observations, morphological descriptions were given for the species, correlating it with the measured genome weights. New ploidies were inferred for *Gagea villosa* which, apart from 14 diploids, had six plants with an inferred tetraploid amount of DNA and for *G. pratensis* that was found to have, apart from the tetraploid, the hexaploid and the very common pentaploid accessions, also a genome size inferring decaploidy.

## Methods

### Plant material

**Fig. 2:**
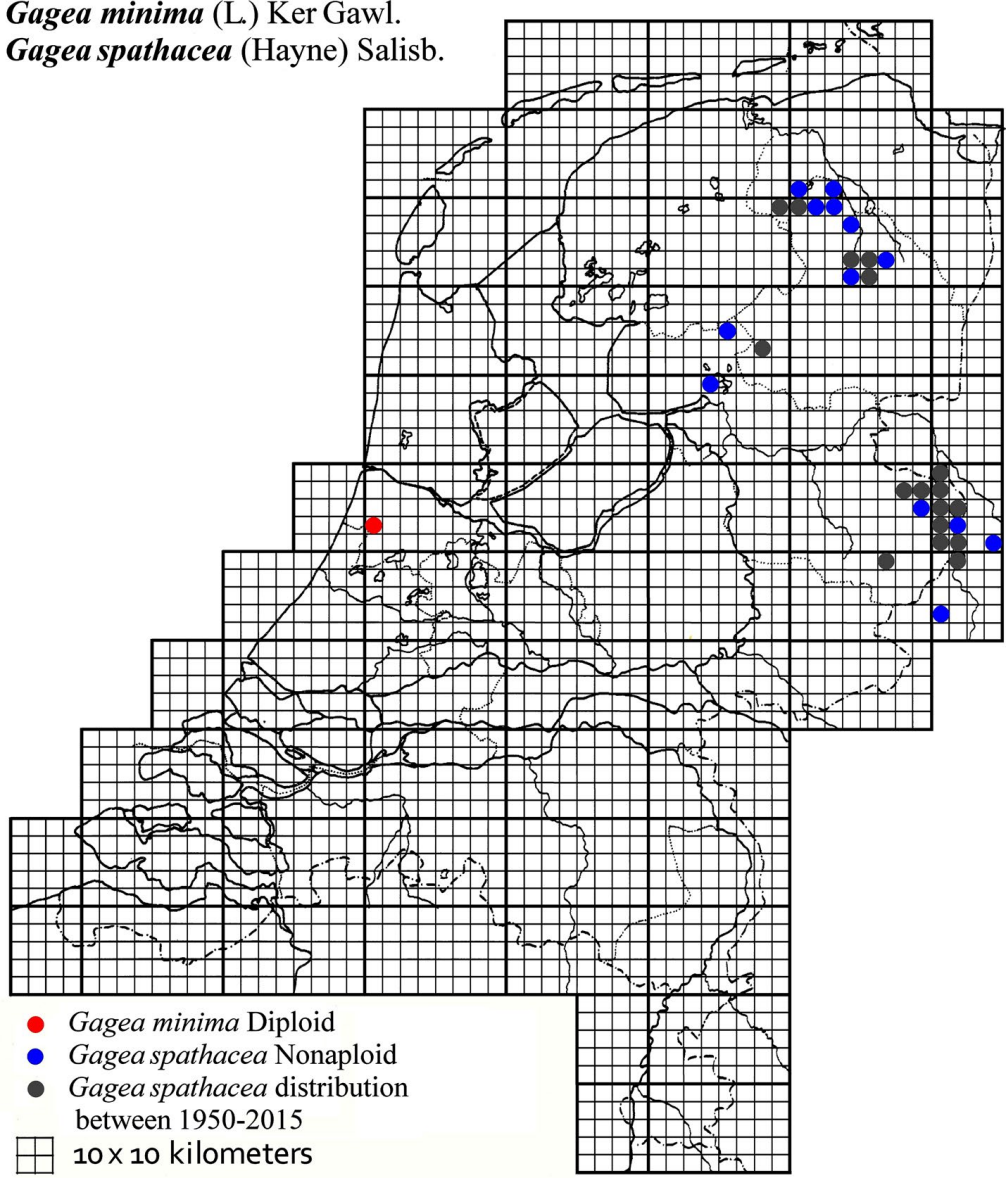
The distribution of *Gagea minima* and *Gagea spathacea* in the Netherlands.

**Fig. 3:**
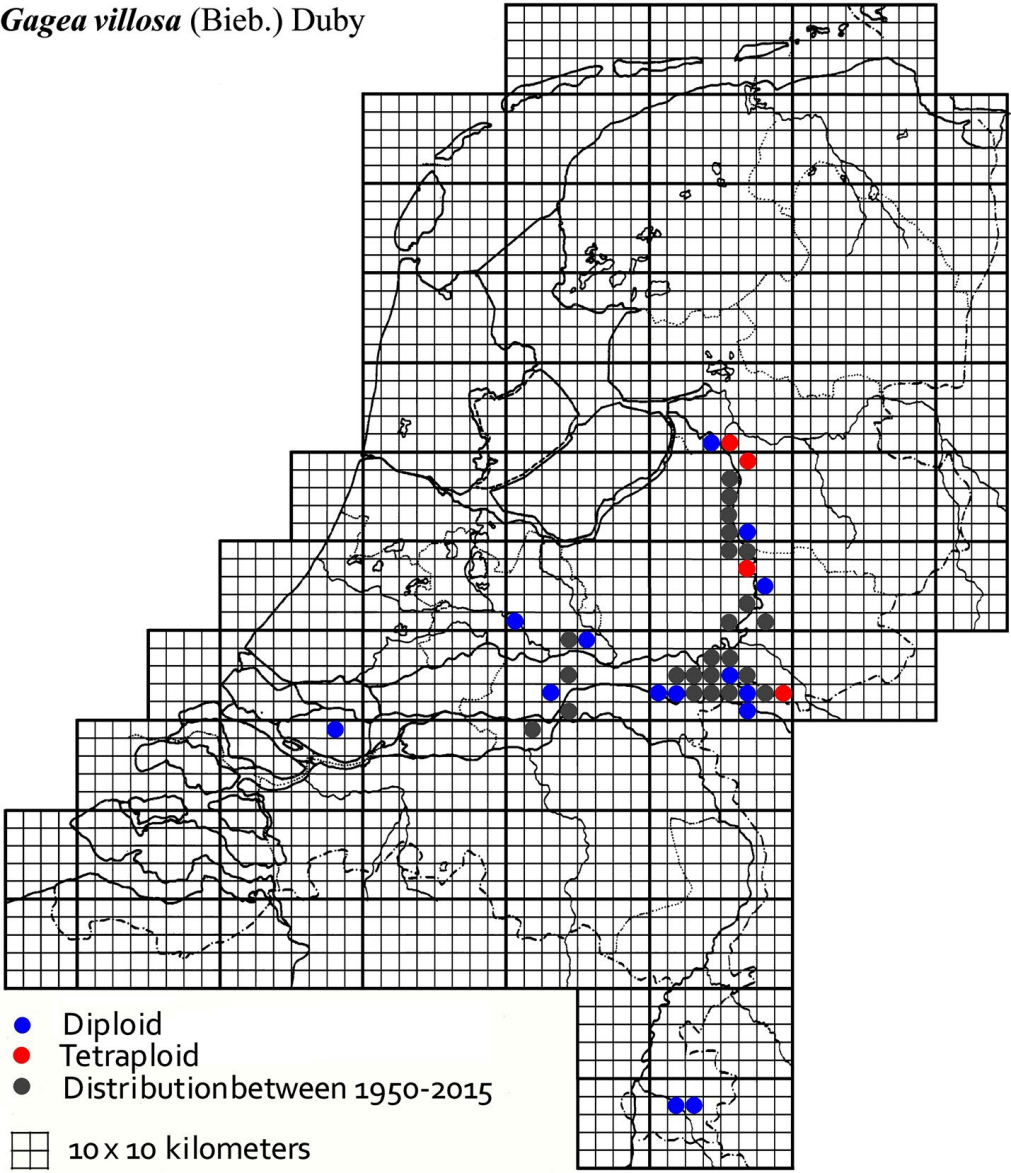
The distribution of *Gagea villosa* in the Netherlands.

**Fig. 4:**
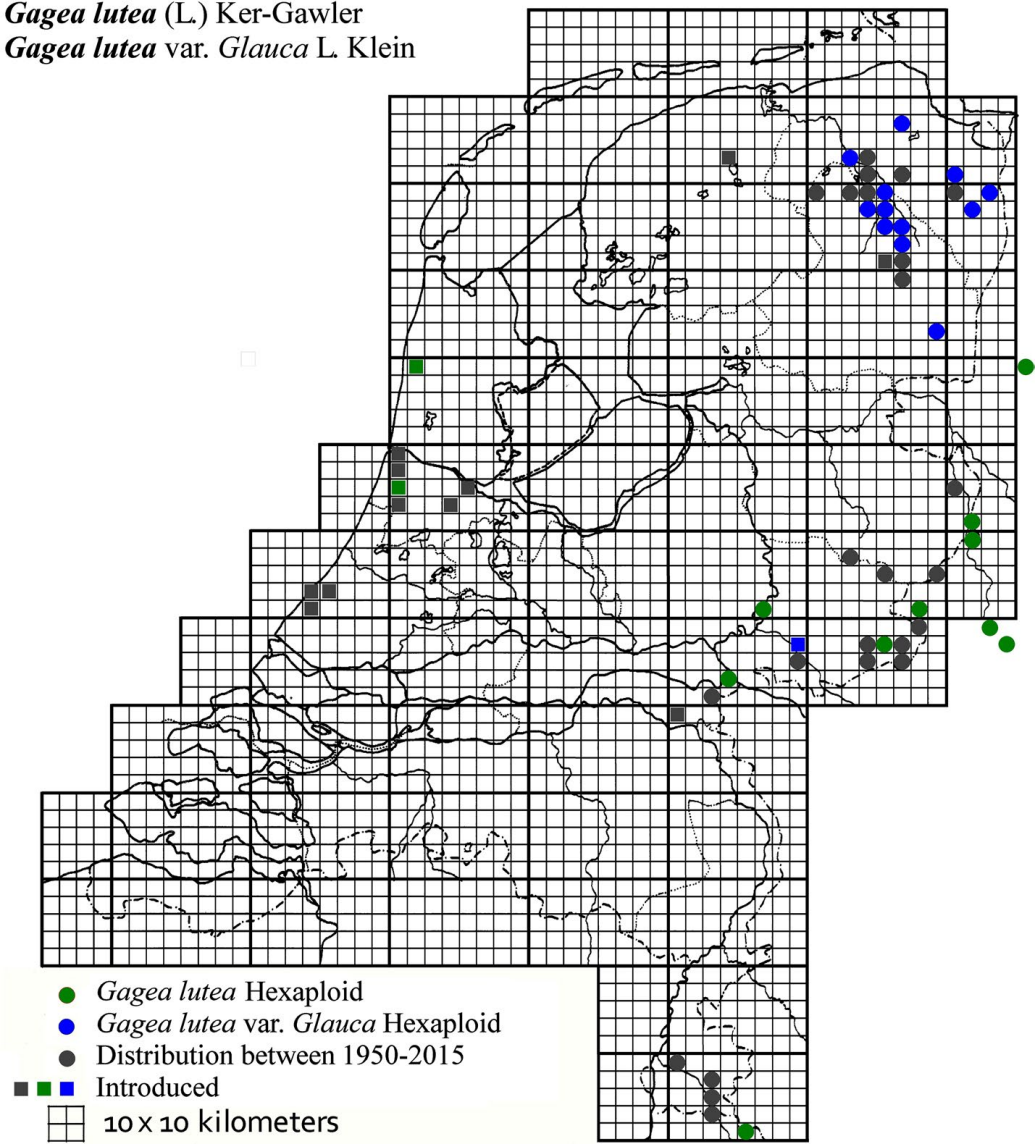
The distribution of *Gagea lutea* and var. *glauca* in the Netherlands.

**Fig. 5:**
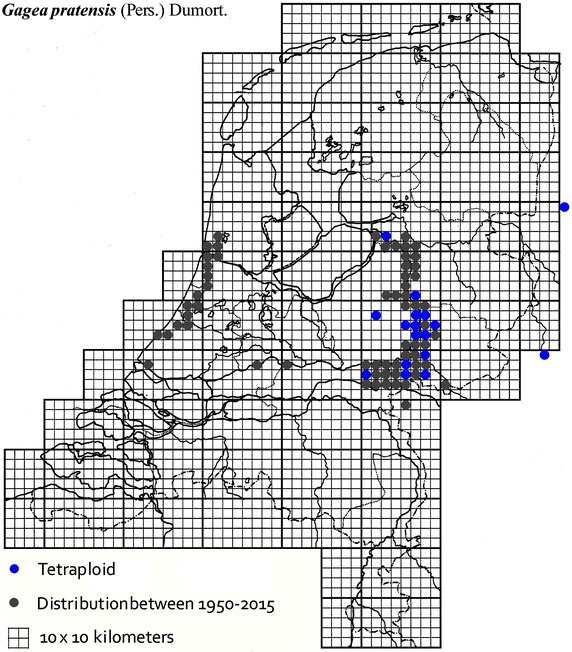
The distribution of tetraploid *Gagea pratensis* in the Netherlands.

**Fig. 6:**
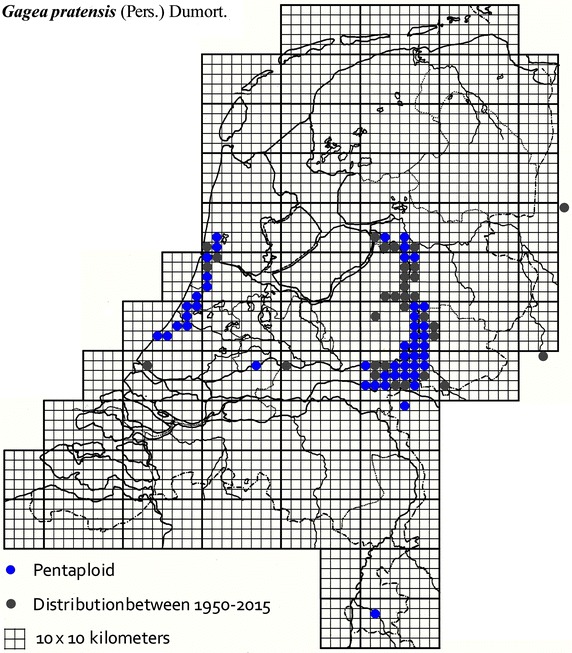
The distribution of pentaploid *Gagea pratensis* in the Netherlands.

**Fig. 7:**
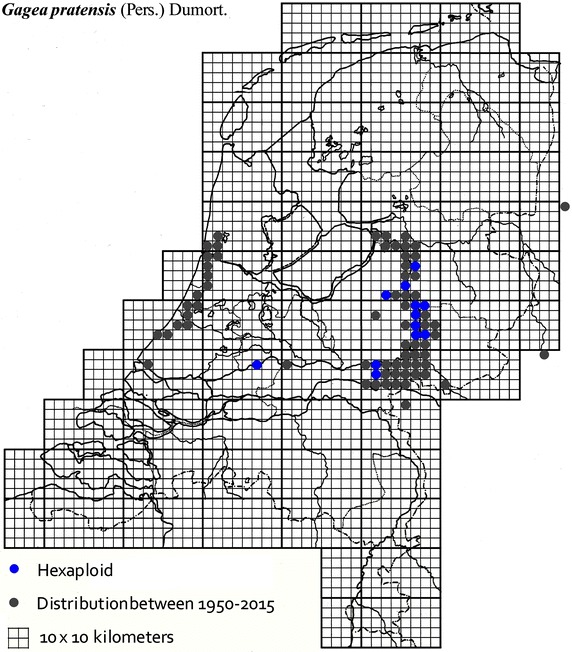
The distribution of hexaploid *Gagea pratensis* in the Netherlands.

**Fig. 8:**
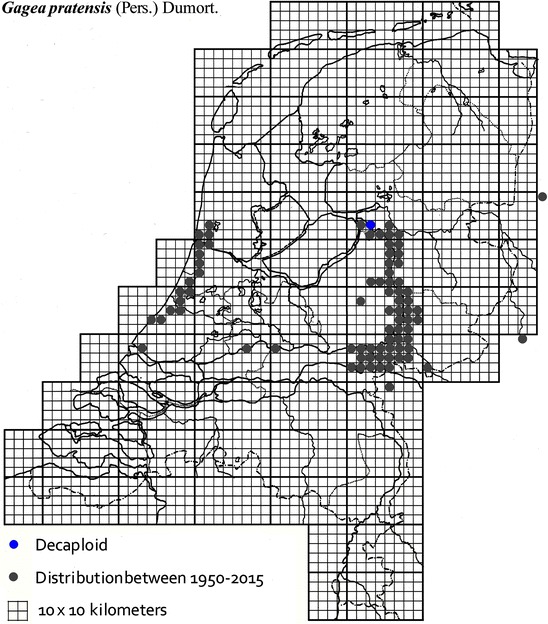
The distribution of decaploid *Gagea pratensis* in the Netherlands.

The plant material used in this study was collected from locations across The Netherlands and Germany as described in Table 1. It was mainly obtained from B. te Linde, Stichting Berglinde, Babberich and a few from L. Duistermaat from NCB Naturalis, Leiden, The Netherlands. Further material came from T. Pfeiffer from the Ernst-Moritz-Arndt-University of Greifswald, Germany. The German plants, supplied with chromosomes counts, were used to infer the ploidy of the Dutch plants. Material of known origin was used. Vouchers will be lodged in the Herbarium of Naturalis Leiden (L). Figures 2, 3, 4, 5, 6, 7, 8 show the spread of the taxa in the Netherlands.

**Table 1:** Accessions of *Gagea* from The Netherlands and Germany (in italics), with their nuclear DNA content, inferred ploidy, average, standard deviations and localities

Species	pg/2C	Average	stdev	Locality	coll. nr	Locality (counties NL)
*Gagea minima* diploid						
*Gagea minima*	14.8	14.9	0.6	Leyduin, Bloemendaal	BtL11-0092	N Holland
*Gagea minima*	14.9			Leyduin, Bloemendaal	L Duistermaat	N Holland
*Gagea villosa* diploid						
*Gagea villosa*	16.5	17.0	0.4	Doddendaal	BtL11-053	Gelderland
*Gagea villosa*	16.7			Deventer	BtL11-085	Overijsel
*Gagea villosa*	16.7			Zutphen, Hanzehof	BtL11-003	Gelderland
*Gagea villosa*	16.8			Elten	BtL11-001	*Germany*
*Gagea villosa*	16.8			Eys	BtL11-002	Limburg
*Gagea villosa*	16.8			Zevenaar	BtL11-084	Gelderland
*Gagea villosa*	16.9			Zeist	BtL11-037	Utrecht
*Gagea villosa*	17.0			Lent	BtL11-055	Gelderland
*Gagea villosa*	17.0			Valkenburg	BtL11-077	Limburg
*Gagea villosa*	17.2			Zevenaar	BtL11-078	Gelderland
*Gagea villosa*	17.3			Zalk	BtL11-017	Overijsel
*Gagea villosa*	17.4			Mijnsherenland	BtL14-002	Gelderland
*Gagea villosa*	17.3			Spijk	BtL14-003	Gelderland
*Gagea villosa*	17.2			Amerongen	BtL14-004	Utrecht
*Gagea villosa* tetraploid						
*Gagea villosa*	31.5	32.3	0.7	Heelsum	BtL11-052	Gelderland
*Gagea villosa*	31.7			Zwolle, Nahuysplein 1	BtL11-016	Overijsel
*Gagea villosa*	31.8			Zwolle, Zandhove	BtL13-079	Overijsel
*Gagea villosa*	32.6			Bussloo, begraafplaats t.o Zutphenboer	BtL13-088	Gelderland
*Gagea villosa*	32.8			Zwolle, Nahuysplein	BtL13-080	Overijsel
*Gagea villosa*	33.2			Azewewijn, kerkhof	BtL13-102	Gelderland
*Gagea pratensis* tetraploid						
*Gagea pratensis*	32.1	32.8	0.4	Deventer	BtL11-085	Overijsel
*Gagea pratensis*	32.1			Kootwijk	BtL11-073	Gelderland
*Gagea pratensis*	32.2			Almen 2	BtL11-066	Gelderland
*Gagea pratensis*	32.2			Legden	BtL13-028	*Germany*
*Gagea pratensis*	32.3			Almen 1	BtL11-040	Gelderland
*Gagea pratensis*	32.3			Rhienderen 1	BtL11-067	Gelderland
*Gagea pratensis*	32.4			Suderas, Wichmond	BtL13-053	Gelderland
*Gagea pratensis*	32.4			Bussloo, begraafplaats t.o Zutphenboer	BtL13-087	Gelderland
*Gagea pratensis*	32.5			Babberich	BtL11-014	Gelderland
*Gagea pratensis*	32.6			Voorst	BtL11-062	Gelderland
*Gagea pratensis*	32.7			Gietelo	BtL13-002	Gelderland
*Gagea pratensis*	32.7			Ravenswaarden	BtL13-009	Gelderland
*Gagea pratensis*	32.7			Keppel, klein	BtL13-050	Gelderland
*Gagea pratensis*	32.8			Almen	BtL11-012	Gelderland
*Gagea pratensis*	32.9			Trent 2	Tre11-xx	*Germany*
*Gagea pratensis*	32.9			Gingst	Gin11-xx	*Germany*
*Gagea pratensis*	32.9			Meppen, stadswal	BtL13-077	*Germany*
*Gagea pratensis*	33.0			Subzow	Sub11-18	*Germany*
*Gagea pratensis*	33.0			Elbe Jasebeck	BtL13-043	*Germany*
*Gagea pratensis*	33.0			Deventer, Brinkgrave	BtL13-084	Overijsel
*Gagea pratensis*	33.1			Almen 3	BtL11-074	Gelderland
*Gagea pratensis*	33.2			Zirchow	Zir11-xx	*Germany*
*Gagea pratensis*	33.2			Deventer, Brinkgrave	BtL13-015	Overijsel
*Gagea pratensis*	33.3			Beek	BtL11-045	Gelderland
*Gagea pratensis*	33.3			Klarenbeek	BtL11-061	Gelderland
*Gagea pratensis*	33.3			Trent	Tre11-xx	*Germany*
*Gagea pratensis*	33.3			Kampens dl	BtL13-035	Overijsel
*Gagea pratensis*	33.4			Gorssel, oud kerkhof	BtL13-083	Gelderland
*Gagea pratensis*	33.7			Bingerden 1a	BtL11-075	Gelderland
*Gagea pratensis*	33.7			Gietelo	BtL13-002	Gelderland
*Gagea pratensis* pentaploid						
*Gagea pratensis*	37.9	39.9	0.5	Cortenoever 1	BtL11-087	Gelderland
*Gagea pratensis*	37.9			Leyduin	BtL11-041	N. Holland
*Gagea pratensis*	38.3			Ressen	BtL11-056	Gelderland
*Gagea pratensis*	38.5			Bingerden 2	BtL11-094	Gelderland
*Gagea pratensis*	38.5			Bemmel 1	BtL11-033	Gelderland
*Gagea pratensis*	38.6			Zwolle 2	BtL11-015	Overijsel
*Gagea pratensis*	38.6			Hattem, Heezenberg	BtL13-089	Gelderland
*Gagea pratensis*	38.7			Angerlo	BtL11-027	Gelderland
*Gagea pratensis*	38.7			Ravenswaarden	BtL11-058	Gelderland
*Gagea pratensis*	38.7			Den Haag, Westerduinpark	BtL13-031	Z. Holland
*Gagea pratensis*	38.8			Castricum, kerkhof	BtL13-085	N. Holland
*Gagea pratensis*	38.9			Didam	BtL13-048	Gelderland
*Gagea pratensis*	38.9			Zwolle, Vecht	BtL13-081	Overijsel
*Gagea pratensis*	39.0			Loil	BtL11-024	Gelderland
*Gagea pratensis*	39.0			Angerlo	BtL11-026	Gelderland
*Gagea pratensis*	39.0			Babberich 2	BtL11-072	Gelderland
*Gagea pratensis*	39.0			Den Haag, Marlot	BtL13-032	Z. Holland
*Gagea pratensis*	39.0			Lisse, Huis te Spekke	BtL13-024	Z. Holland
*Gagea pratensis*	39.1			Haarlem 1a	BtL11-039	N. Holland
*Gagea pratensis*	39.1			Leiden, Rhijnhof	BtL13-042	Z. Holland
*Gagea pratensis*	39.1			Keppel, groot	BtL13-051	Gelderland
*Gagea pratensis*	39.2			Bemmel	BtL11-032	Gelderland
*Gagea pratensis*	39.2			Velp 2	BtL11-029	Gelderland
*Gagea pratensis*	39.2			Bemmel 2	BtL11-032	Gelderland
*Gagea pratensis*	39.2			Noordwijk, Gooweg	BZ13-023	Z. Holland
*Gagea pratensis*	39.2			Haarlem, Schootersingel	BtL13-036	N. Holland
*Gagea pratensis*	39.2			Driehuis begraafplaats	BtL13-037	N. Holland
*Gagea pratensis*	39.2			Huize Baak	BtL13-052	Gelderland
*Gagea pratensis*	39.2			Deventer, Drouwelerkolk	BtL13-013	Overijsel
*Gagea pratensis*	39.2			Hattemerwaard	BtL13-093	Gelderland
*Gagea pratensis*	39.2			Velzen, Beeckenstein	BtL13-101	N. Holland
*Gagea pratensis*	39.3			Huissen, pastorie	BtL13-040	Gelderland
*Gagea pratensis*	39.3			Voorhout	BtL13-022	Z. Holland
*Gagea pratensis*	39.3			Eldrik	BtL13-049	Gelderland
*Gagea pratensis*	39.3			Kampen, stadswal	BtL13-033	Overijsel
*Gagea pratensis*	39.3			Mehr	BtL13-027	*Germany*
*Gagea pratensis*	39.4			Bingerden 1b	BtL11-076	Gelderland
*Gagea pratensis*	39.4			Kampen 1a	BtL11-019	Overijsel
*Gagea pratensis*	39.4			Brummen 1	BtL11-068	Gelderland
*Gagea pratensis*	39.4			Warmond	BtL13-021	Z. Holland
*Gagea pratensis*	39.5			Fromberg	BtL11-093	Limburg
*Gagea pratensis*	39.5			Oud-Zevenaar	BtL11-023	Gelderland
*Gagea pratensis*	39.5			Elten	Elt11-xx	*Germany*
*Gagea pratensis*	39.5			Drempt	BtL11-042	Gelderland
*Gagea pratensis*	39.5			Zutphen, Hanzehof	BtL13-001	Gelderland
*Gagea pratensis*	39.5			Zutphen, kanaal 16 jan 2013	BtL13-003	Gelderland
*Gagea pratensis*	39.5			Epse	BtL13-005	Gelderland
*Gagea pratensis*	39.5			Wassenaar	BtL13-006	Z. Holland
*Gagea pratensis*	39.5			Sassenheim	BtL13-010	Z. Holland
*Gagea pratensis*	39.5			Zutphen, De Hoven	BtL13-011	Gelderland
*Gagea pratensis*	39.5			OegstgeesT	BtL13-012	Z. Holland
*Gagea pratensis*	39.5			IJmuiden berm	BtL13-038	N. Holland
*Gagea pratensis*	39.5			Velzen, Kanaalweg	BtL13-086	N. Holland
*Gagea pratensis*	39.5			Achthoven	BtL14-005	Gelderland
*Gagea pratensis*	39.6			Haarlem	BtLs.n.	N. Holland
*Gagea pratensis*	39.6			Hoog-Keppel	BtL11-043	Gelderland
*Gagea pratensis*	39.6			Zwolle, Zandhove	BtL13-082	Overijsel
*Gagea pratensis*	39.6			Velzen, kerkhof	BtL13-100	N. Holland
*Gagea pratensis*	39.6			sdl Zutphen, Hanzehof	BtL14-08	Gelderland
*Gagea pratensis*	39.6			sdl Zutphen, Hanzehof	BtL14-008	Gelderland
*Gagea pratensis*	39.7			Zutphen	Btl11-022	Gelderland
*Gagea pratensis*	39.7			Cortenoever 2	Btl11-020	Gelderland
*Gagea pratensis*	39.8			Beverwijk, Scheybeek	BtL13-039	N. Holland
*Gagea pratensis*	39.8			Zwolle, Engelse werk	BtL13-078	Overijsel
*Gagea pratensis*	39.9			Hummelo	BtL11-044	Gelderland
*Gagea pratensis*	39.9			Leiden, Rhynhof	BZ12-01	Z. Holland
*Gagea pratensis*	39.9			Den Haag 2	BtL11-070	Z. Holland
*Gagea pratensis*	40.0			sdl Zutphen, Hanzehof	BtL14-008	Gelderland
*Gagea pratensis*	40.0			sdl Zutphen, Hanzehof	BtL14-008	Gelderland
*Gagea pratensis*	40.1			Rhienderen 2	BtL11-067	Gelderland
*Gagea pratensis*	40.1			Zutphen, Hanzehof	BtLs.n.	Gelderland
*Gagea pratensis*	40.2			Cortenoever 3	BtL11-021	Gelderland
*Gagea pratensis*	40.2			Weurt	BtL11-054	Gelderland
*Gagea pratensis*	40.2			Heelsum, kerk	BtL14-011	Gelderland
*Gagea pratensis*	40.3			Brummen 2	BtL11-071	Gelderland
*Gagea pratensis*	40.3			sdl Zutphen, zwembad	BtL14-009	Gelderland
*Gagea pratensis*	40.4			Doesburg	BtL11-028	Gelderland
*Gagea pratensis*	40.4			Middachten	BtL11-046	Gelderland
*Gagea pratensis*	40.4			parent Zutphen, zwembad	BtL14-010	Gelderland
*Gagea pratensis*	40.5			Spankeren	Btl11-060	Gelderland
*Gagea pratensis*	40.6			Olburgen	Btl11-047	Gelderland
*Gagea pratensis*	40.6			Steenderen	Btl11-048	Gelderland
*Gagea pratensis*	40.7			Zutphen, begraaf plaats	BtL13-054	Gelderland
*Gagea pratensis*	40.9			Groessen	BtL11-035	Gelderland
*Gagea pratensis*	40.9			Oud-Zevenaar	BtL11-057	Gelderland
*Gagea pratensis* hexaploid						
*Gagea pratensis*	43.8	45.6	1.1	Zirchow/U 2	ZiU11-xx	*Germany*
*Gagea pratensis*	44.0			Trent 2	Tre11-xx	*Germany*
*Gagea pratensis*	44.3			Vaassen, Canneburg 194/478	BtL11-095	Limburg
*Gagea pratensis*	45.0			Bronkhorst, slotheuvel	BtL11-049	Gelderland
*Gagea pratensis*	45.1			Deventer, De Worp	BtL13-004	Overijsel
*Gagea pratensis*	45.1			Culemborg, Stroomrug	BtL13-008	Gelderland
*Gagea pratensis*	45.2			Brummen, Engelenburg	BtL13-017	Gelderland
*Gagea pratensis*	45.3			Oosterbeek, gazon	BtL11-051	Gelderland
*Gagea pratensis*	45.4			Meppen begraafplaats	BtL13-072	*Germany*
*Gagea pratensis*	45.4			Deventer, Blauwijk	BtL13-014	Overijsel
*Gagea pratensis*	45.4			Marle, uiterwaardgrasland	BtL13-090	Overijsel
*Gagea pratensis*	45.7			Brummen, Ganzenei	BtL13-091	Gelderland
*Gagea pratensis*	45.8			Meppen stadswal	BtL13-071	*Germany*
*Gagea pratensis*	46.0			Elst, Johan de Wittstraat	BtL11-030	Gelderland
*Gagea pratensis*	46.0			Altenkirchen	Alt11-19	*Germany*
*Gagea pratensis*	46.1			Olst, gementehuis	BtL13-016	Overijsel
*Gagea pratensis*	46.6			Ravenswaarden	BtL13-099	Gelderland
*Gagea pratensis*	47.2			Empe	BtL11-063	Gelderland
*Gagea pratensis*	47.4			Brummen 3	BtL11-064	Gelderland
*Gagea pratensis*	47.9			Brummen 4	BtL11-069	Gelderland
*Gagea pratensis* decaploid						
*G. pratensis*	75.0	75.8	1.1	Kampen 1b	BtL11-018	Overijsel
*G. pratensis*	76.6			Kampen 1c	BtL11-060	Overijsel
*Gagea* × *megapolitana* hexaploid						
*Gagea* × *megapolitana*	46.7	46.8	0.1	Gingst 2	Gin11-xx	*Germany*
*Gagea* × *megapolitana*	46.8			Meppen	BtL13-071	*Germany*
*Gagea lutea* hexaploid						
*Gagea lutea*	41.7	42.7	0.6	Trent 1	DEs.n.	*Germany*
*Gagea lutea*	41.9			Haarlem, Spaem en Hout	BtL11-039	N. Holland
*Gagea lutea*	42.1			Epe, Dinkel	BtL13-030	*Germany*
*Gagea lutea*	42.1			Vreden, Berkel	BtL13-029	*Germany*
*Gagea lutea*	42.1			Miste	BtL13-103	Gelderland
*Gagea lutea*	42.3			Roden	BtL11-081	Drenthe
*Gagea lutea*	42.3			Kelmis, Hohntal	BtL11-079	Belgie
*Gagea lutea*	42.4			Zuidlaren	BtL11-089	Drenthe
*Gagea lutea*	42.4			Griebenow	DE-s.n.	*Germany*
*Gagea lutea*	42.6			Meppen, stadswal	BtL13-070	*Germany*
*Gagea lutea*	42.6			Bron Berkle	Bbe11-xx	*Germany*
*Gagea lutea*	42.6			Bredevoort	BtL11-011	Gelderland
*Gagea lutea*	42.8			Haarlem 1c	BtL11-038	N. Holland
*Gagea lutea*	42.8			D Meppen	BtL13-026	*Germany*
*Gagea lutea*	42.8			Millingerwaard	BtL14-001	Z. Holland
*Gagea lutea*	42.9			Gesher	Ges11-xx	*Germany*
*Gagea lutea*	42.9			Haarlem, Spaem en Hout	BtL11-38	N. Holland
*Gagea lutea*	42.9			Elbe, Jasebeck	BtL13-044	*Germany*
*Gagea lutea*	43.2			Aerdenhout	BtL13-066	N. Holland
*Gagea lutea*	43.4			Winterswijk, Vreehorst weg	BtL13-055	Gelderland
*Gagea lutea*	43.8			Bronkhorst	BtL11-005	Gelderland
*Gagea lutea*	43.9			Schoorl	BtL13-094	N. Holland
*Gagea lutea* var. *glauca*	41.9	42.3	0.3	Anloo, grasland	BtL13-064	Drenthe
*Gagea lutea* var. *glauca*	41.9			Veenhof, Berm	BtL13-057	Drenthe
*Gagea lutea* var. *glauca*	42.0			Groningen, Noorderplantsoen	BtL13-045	Groningen
*Gagea lutea* var. *glauca*	42.1			Zeegse	BtL11-090	Drenthe
*Gagea lutea* var. *glauca*	42.1			Bellingwolde, berm	BtL13-067	Groningen
*Gagea lutea* var. *glauca*	42.2			Midwolda, Ennemaborg	BtL13-047	Groningen
*Gagea lutea* var. *glauca*	42.2			Eext Berm	BtL13-058	Drenthe
*Gagea lutea* var. *glauca*	42.2			Westeresch	BtL11-080	Drenthe
*Gagea lutea* var. *glauca*	42.3			Sterrenbospark	BtL11-088	Groningen
*Gagea lutea* var. *glauca*	42.3			Gieten	BtL11-009	Drenthe
*Gagea lutea* var. *glauca*	42.3			Doetinchem, Zumpe 16 jan 2013	BtL13-007	Gelderland
*Gagea lutea* var. *glauca*	42.4			Midlaren	BtL13-059	Drenthe
*Gagea lutea* var. *glauca*	42.8			Wedde, Huis Te Wedde, onder linde	BtL13-069	Groningen
*Gagea lutea* var. *glauca*	42.8			Appingedam, Ekenstein	BtL13-046	Groningen
*Gagea lutea* var. *glauca*	43.0			Eext, Brink	BtL13-056	Drenthe
*Gagea lutea* var. *glauca*	42.9			Naumburg	BtL14-012	*Germany*
*Gagea* × *pomeranica* pentaploid						
*Gagea* × *pomeranica*	34.2	34.9	0.6	Vitense	Vit11-11	*Germany*
*Gagea* × *pomeranica*	34.3			Zirchow/U	ZIU11-xx	*Germany*
*Gagea* × *pomeranica*	34.3			Zirchow/U 2	ZIU11-xx	*Germany*
*Gagea* × *pomeranica*	34.4			W. Baggendorf	WBa11-15	*Germany*
*Gagea* × *pomeranica*	34.8			Zirchow/U 2	ZiU11-xx	*Germany*
*Gagea* × *pomeranica*	35.1			Semlow 2	Sem11-xx	*Germany*
*Gagea* × *pomeranica*	35.2			Semlow	Sem11-xx	*Germany*
*Gagea* × *pomeranica*	35.2			Poseritz	Pos11-12	*Germany*
*Gagea* × *pomeranica*	35.3			Semlow 2	Sem11-xx	*Germany*
*Gagea* × *pomeranica*	35.5			Zirkow/R	ZiR11-12	*Germany*
*Gagea* × *pomeranica*	36.0			Semlow	Sem11-xx	*Germany*
*Gagea spathacea* nonaploid						
*Gagea spathacea*	45.4	46.7	0.8	Ootmarsum, de Voort	BtL13-063	Z. Holland
*Gagea spathacea*	45.4			Zeegse	BtL11-091	Drenthe
*Gagea spathacea*	45.5			Losser	BtL11-082	Overijsel
*Gagea spathacea*	45.9			Samerot, eiken-haagbeukenbos	BtL13-074	*Germany*
*Gagea spathacea*	46.1			Vasse, beekoever	BtL13-076	Overijsel
*Gagea spathacea*	46.1			Brummen, Ganzenei, stroomrug	BtL13-092	Gelderland
*Gagea spathacea*	46.3			Amen	BtL14-006	Drenthe
*Gagea spathacea*	46.4			Bentheim, langs pad	BtL13-073	*Germany*
*Gagea spathacea*	47.0			Roden, Havezate	BtL11-081	Drenthe
*Gagea spathacea*	47.0			Peizermade, bosrand	BtL13-096	Drenthe
*Gagea spathacea*	47.2			Roden, Havezate	BtL13-097	Drenthe
*Gagea spathacea*	47.3			Wüllen, eiken-haagbeukenbos	BtL13-068	*Germany*
*Gagea spathacea*	47.5			Nietap	BtL13-098	Drenthe
*Gagea spathacea*	47.6			Bentheim, gazon	BtL13-041	*Germany*
*Gagea spathacea*	47.7			Ootmarsum, weilandrand	BtL13-075	Overijsel
*Gagea spathacea*	47.9			Varik, eikenbos	BtL13-065	Drenthe
*Gagea spathacea*	48.1			Peize	BtL13-095	Drenthe

All were measured against *Agave americana*, but for *G. minima* and *G. villosa* *A. attenuta* was used.

*BtL* B. te Linde, *stdev* standard deviation, *coll.nr* collection number.

### Flow cytometric measurement of DNA 2C-value

For the isolation of nuclei, a few cm of leaf or a single bulbil was chopped together with a piece of *Agave americana* L. ‘Aureomarginata’ or *Agave attenuata* L. as an internal standard (see below). The chopping was done with a new razor blade in a Petri dish in 0.25 ml nuclei-isolation buffer to which 0.25 mg RNase/ml was added (Zonneveld and van Iren 2001). After adding 1.75 ml propidium iodide solution (50 mg PI/l in isolation buffer) the suspension with nuclei was filtered through a 20 μm nylon filter. The fluorescence of the nuclei at 585 nm was measured half an hour and 1 h after addition of propidium iodide excitation, using a BD Accuri C6 flow cytometer equiped with a 488 nm laser suitable for propidium iodide. Data were analyzed by means of BD Accuri Cflow Plus software provided by the supplier. Plots were first gated to exclude debris on a scatter diagram (Fl2-A vs FL1-A) and counted against FL2-A on a logarithmic scale. The 2C DNA content of the sample was calculated as the sample peak mean, divided by the *Agave* peak mean, and multiplied with the amount of DNA of the *Agave* standard. Two different samples, with each at least 5,000 nuclei, were measured twice for each clone. Most histograms revealed a Coefficient of Variation of less than 5%. The standard deviation was calculated for the DNA content of each species, using all relevant measurements.

### Internal standard and absolute DNA content values

When measuring nuclear DNA content by means of flow cytometry, it is necessary to chop tissue from the plant of interest together with an internal standard. This standard must be as close as possible to the plants of interest and not overlap with the ploidy area of interest. If they are too close together the peak values interfere with each other. Linearity is checked by comparing the different ploidies as found within leaves and roots of many plants. In this way, variation in signal intensities due to staining kinetics, to light absorption and quenching by sample components, as well as to instrument and other variables, is reduced to a minimum. *Agave americana* was chosen as internal standard for *Gagea*. For *Gagea minima* and *G. villosa*, with 2C-values that more or less coincided with *Agave americana*, *Agave attenuata* was used. *Agave* is available year-round, does not mind several weeks without water and, being a large plant, a single specimen can serve a lifetime, thereby further reducing variation in readings. It also has a low background in propidium iodide measurements, and show a single G_0_ peak, almost lacking G_2_ arrest.

Fresh male human leucocytes [2C = 7.0 pg; 1 pg = 10^−12^ g = 0.978 × 10^9^ base pairs (Doležel et al. 2003)] were chosen as primary standard (Tiersch et al. 1989). This yields 2C = 15.9 pg for nuclei of *Agave americana* L. and 8.0 pg for *A. attenuata*. Based on a published male human genome size of 6.294 × 10^9^ base pairs the nucleus was calculated as containing 6.436 pg (Doležel et al. 2003). However this is based on a human sequence where the size of the very large repeat sequences could not accurately be determined. So in the end the genome size could be closer to 7 pg than now envisioned.

## Results

### General

Morphologically the species of *Gagea* are rather difficult to differentiate. They are all small bulbous plants with grass like leaves and mostly yellow flowers. Moreover they are visible above the soil surface only about 2 months a year in early spring. The Dutch *Gagea* can be divided over four out of 12–14 different sections. *G. lutea*, *G.* × *pomeranica* and *G. pratensis* belong to section Gagea *whereas G. minima*, *G. villosa* and *G. spathacea* each belong to a separate section. Gageas have been measured from 186 localities in The Netherlands (Tables 1, 2) and they are compared with 41 accessions from Germany. They are shown to comprise six taxa with several inferred ploidies.

**Table 2:** Summary of genome sizes in pg (2C), number of accessions and inferred chromosome numbers of species of Dutch and German gageas

Species	Average pg/2C	Inferred ploidy	Chromosome number	Number of accessions
*Gagea minima* (L.) Ker Gawl.	14.9	Diploid	2x = 24	2
*Gagea villosa* (M.Bieb.) Sweet	17.0	Diploid	2x = 24	14
*Gagea villosa* (M.Bieb.) Sweet	32.3	Tetraploid	4x = 48	6
*Gagea lutea* (L.) Ker Gawl.	42.7	Hexaploid	6x = 72	22
*Gagea lutea* var. *glauca* L.Klein	42.3	Hexaploid	6x = 72	16
*Gagea spathacea* (Hayne) Salisb.	46.7	Nonaploid	9x = 108	17
*Gagea pratensis* (Pers.) Dumet.	32.8	Tetraploid	4x = 48	30
*Gagea pratensis* (Pers.) Dumet.	39.9	Pentaploid	5x = 60	85
*Gagea pratensis* (Pers.) Dumet.	45.6	Hexaploid	6x = 72	20
*Gagea pratensis* (Pers.) Dumet.	75.8	Decaploid	10x = 120	2
*G.* × *pomeranica* R.Ruthe	34.9	Hexaploid	5x = 60	11
*G.* × *megapolitana* Henker	46.8	Hexaploid	6x = 72	2

The term ‘inferred ploidy’ indicates that the ploidy is derived from the genome size and not based on chromosome counts. It is preferred to the proposed term ‘DNA ploidy’ (Suda et al. 2006) as this seems more ambiguous. Inferred decaploidy is found for the first time in *G. pratensis.* The hybrid *G.* × *megapolitana*, is only collected in Germany so far. The largest genome contains roughly 60 × 10^9^ more base pairs than the smallest. A difference of 1 pg amounts to a difference of nearly 1 × 10^9^ base pairs, so far exceeds a single taxonomic character.

### *Gagea minima* (L.) Ker Gawl.-section Minimae

*Gagea minima* is a small plant with 1 (or 2) narrow 2–3(5) mm wide leaves and 1–3 flowers per scape. *G. minima* with 2C = 14.9 pg from two localities, together with *G. villosa*, are the only two inferred diploid species found in The Netherlands.

### *Gagea villosa* (M.Bieb.) Sweet-section Didymobolbos

*Gagea villosa* is a hairy, largely sterile plant with numerous bulbils. Fourteen accessions of *G. villosa* from the Dutch provinces of Gelderland, Overijsel and Zuid-Holland are inferred to be diploid with 16.9 pg. Six accessions of *G. villosa* are inferred to be tetraploid with on average 32.3 pg. This is based on the basic value of 7–8 pg as in the other species (except *G. spathacea*) and the published counts of 24 and 48 chromosomes (http://www.tropicos.org/gagea).

### *Gagea pratensis* (Pers.)Dumort.-section Gagea

**Fig. 9:**
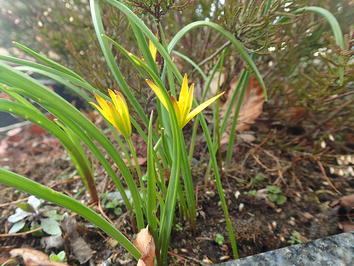
*Gagea pratensis* tetraploid April 6, 2015 in a churchyard in Beek.

**Fig. 10:**
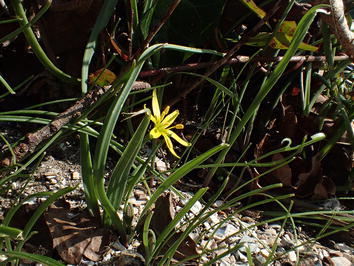
*Gagea pratensis* pentaploid (a) March 17, 2015 in a churchyard in Wassenaar.

**Fig. 11:**
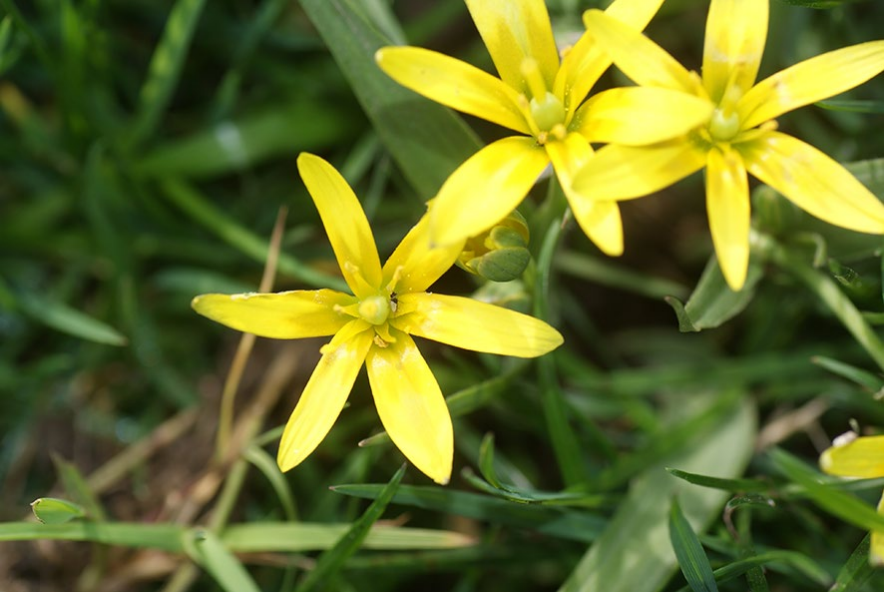
*Gagea pratensis* pentaploid (b) April 3, 2011 in Bingerden.

**Fig. 12:**
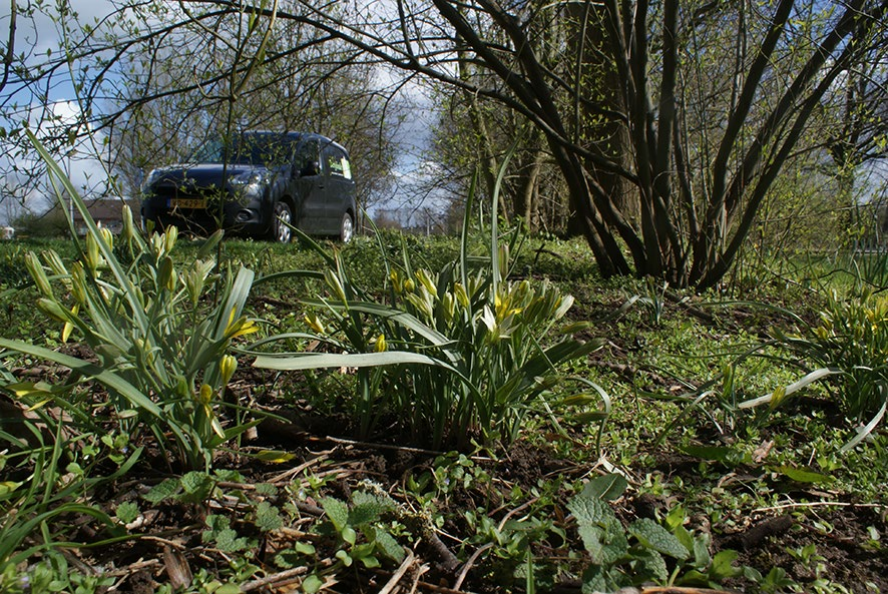
*Gagea pratensis* pentaploid (c) March 23, 2014 in a park in Zutphen.

**Fig. 13:**
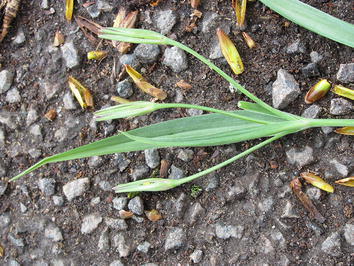
*Gagea pratensis* hexaploid April 22, 2011 in a road verge in Brummen.

*Gagea pratensis* is a glabrous plant with up to four flowers per scape. Characteristic are the two nude egglike, horizontal bulbils. *Gagea pratensis* can be found in The Netherlands with four different inferred ploidies. They can be recognized in that the tetraploid has the leaf sheath circling the stem halfway, the pentaploid three-quarter and the hexaploid and the decaploid completely. They are shown in Figs. 9, 10, 11, 12 and 13. The tetraploids (30 accessions) have a DNA 2C-value (nuclear DNA content) of on average 32.8 pg, the pentaploids (85 accessions) have on average 39.9 pg and the hexaploids (20 accessions) have on average 45.6 pg. The pentaploids could be hybrids between the tetraploid and the hexaploid cytotypes. Even a decaploid with 75.8 pg has been found. The pentaploid form of *G. pratensis* is by far the most common *Gagea* in The Netherlands with 39.5 pg from 85 out of 186 localities. The same ploidy is counted in all 7 populations of *G. pratensis* from Mecklenburg (Germany) (Henker 2005). Therefore it seems most likely that the decaploid plant is derived from the frequently found pentaploid *G. pratensis* that has in this case doubled its genome. As often in polyploids, DNA might have been lost and a similar loss is found in the hexaploid *G. pratensis* but not in the lower ploidies. The inferred decaploid plants have not been reported before for *G. pratensis*. Being pentaploid in most cases, it comes as no surprise that *G. pratensis* is considered to be sterile (van der Meijden 2005). Taxa with anorthoploid chromosome sets often show a highly irregular meiosis. An exception are large plants from Zutphen, NL that are fully fertile and differ morphologically with a large basal leaf and 4–8 flowers to a stem. They have a genome size similar to pentaploid *G. pratensis*, but look more like *G.* × *megapolitana* Henker (Henker 2005). Out of 50 germinated seeds, five seedlings measured from the Zutphen locality had the same genome size as their parents. This is peculiar for a pentaploid. Earlier analysis of seedlings of the triploid Hosta ‘Sum and Substance’ show different, but lower genome sizes in the seedlings (Zonneveld and Pollock 2012). Pfeiffer et al. (2013) report also that some pentaploid populations of *G. pratensis* are partially fertile. None of the calculated genome sizes of the possible hybrids between *G. lutea* and *G. pratensis* would fit the plants from Zutphen. Hence more research is required to explain these results.

### *Gagea lutea* (L.) Ker Gawl.-section Gagea

*Gagea lutea* is a glabrous plant with leaves of more than 1 cm wide and up to seven flowers per stem. The bulbs have a diameter of 0.75–1.5 cm and form numerous bulbils.

Apart from hexaploid *G. pratensis* also *G. lutea* is inferred to be hexaploid with 2C = 42.7 pg, collected in 22 localities. *Gagea lutea* var. *glauca* (a synonym of *G. lutea* (L.)Ker Gawl.) differs in its glaucous leaves, slightly larger petals, lower fertility and the anthropogenic habitats it grows in. The flowering time of the glaucous forms starts about 2 weeks later when transplanted in the garden. *Gagea* var*. glauca* is restricted to the northern part of the Netherlands and is found in localities separate from the green-leaved form. However, with 42.3 pg for 16 different accessions there is no significant difference in genome size.

### *Gagea spathacea*(Hayne) Salisb.-section Spathaceae

*Gagea spathacea* is a glabrous plant with 1–3 flowers per stem and is usually found in fairly moist places*. Gagea spathacea* is only present as a nonaploid plant across (Eastern) Europe (Westergaard 1936; Henker 2005; Pfeiffer et al. 2012). It is observed in about 70 localities in The Netherlands, of which material was collected at 12 localities with an average of 2C = 46.5 pg. This implies a low basic (Cx) value of 5.2, instead of 7.4–8.4 for the other three species. The virtually sterile *G. spathacea* (Pfeiffer et al. 2012) seems to be a nearly monoclonal plant able to occupy a significant range by dispersal of bulbils (Pfeiffer et al. 2011; 2013).

### Hybrid species

Hybridization and polyploidy are amongst the most important evolutionary mechanism in plants. The parents can be deduced by comparing the genome size of possible parents and their offspring. If parents have say 20 and 30 pg then their offspring will mostly have 25 pg. In more complicated allopolyploids the contribution of each parent can often be calculated. In *Gagea* inferred polyploids run from triploid to decaploid (Peruzzi 2003) whereby several species show different ploidies (Henker 2005). Three different hybrids have been described that have the same parents, *G. lutea* and *G. pratensis* but combining different ploidies. These three hybrids are here discussed under the names as found in the literature. They are not found in The Netherlands, but two of the hybrids were obtained from Germany (Table 1). The hybrids mostly occur in anthropogenically disturbed sites like churchyards, parks and marginally used meadows. Their parent species are found in forests (*G. lutea*) and forest edges (*G. pratensis*). Based on maternal inheritance of the plastids *G. pratensis* provide the female gametes for *G.* × *pomeranica* and *G. megapolitana* (Peterson et al. 2009).

### *Gagea* × *pomeranica* R.Ruthe

The pentaploid *G.* × *pomeranica* (R.Ruthe) Henker with two genomes of the tetraploid *G. pratensis* and three genomes of the hexaploid *G. lutea* (Peterson et al. 2009). However, in the case of *G.* × *pomeranica*, 11 accessions were obtained from Germany that had on average a nuclear DNA content of 34.9 pg. This differs considerably (2.2 pg) from the calculated genome size of 37.1 pg, based on the basic values for *G. lutea* and *G. pratensis*. One explanation could be that this hybrid is an old one and has lost DNA. Another possibility is that other species are involved. Pfeiffer et al. (2013) have shown that backcrosses of the hybrid, mostly with the fully fertile hexaploid *G. lutea* as pollen parent are possible. However, backcrosses of *G.* × *pomeranica* (34.9 pg) with *G. lutea* (42.6 pg) might give higher 2C-values not lower, but these were not observed.

### *Gagea* × *marchica* Henker. Kiesew., U.Raabe, Rätzel

Recently another sterile pentaploid hybrid was described as *G. marchica* Henker et al. (2012) It is described as falling morphologically between the pentaploid *G.* (×) *pomeranica* and the hexaploid *G.* (×) *megapolitana* with 57, 59 but probably 60 chromosomes. If it is supposed to be the reversed hybrid (compared to the parents of *G* × *pomeranica)* between hexaploid *G. pratensis* and tetraploid *G. lutea* the problem arises that a tetraploid *G. lutea* has not been reported so far (Pfeiffer et al. 2013).

### *Gagea* × *megapolitana* Henker

A third hybrid with the same parents is the hexaploid *G.* × *megapolitana* Henker with three genomes of the hexaploid *G. pratensis* and three genomes of the hexaploid *G. lutea* (Peterson et al. 2009). It was obtained from two localities in Germany with on average 2C = 46.8 pg. In the world checklist for monocots (Govaerts 2006) *G. megapolitana* is accepted as a species. However Peterson et al. (2009) have clearly shown that it is a hybrid between the hexaploids *G. pratensis* and *G. lutea*. The genome size provides a firm argument for this hybridity and confirm the suggestion of Peterson et al. (2009) for the parents and the ploidy of *G.* × *megapolitana*.

## Conclusions

Five species and different inferred ploidies are recorded for The Netherlands, as summarized in Table 2, some of the latter for the first time. Inferred decaploidy in *G. pratensis* was not demonstrated earlier. *G. minima* has an inferred diploid size. *G. minima* was only recently (1994) recognized as a new species for the Netherlands (Diemeer 2005). It is not clear whether it reached Haarlem by itself or was imported with lime trees from abroad. After all, Linnaeus lived there for 3 years only a kilometer away. The nearest known locality is 300 km away in Germany. *G. pratensi*s is inferred to have four cytotypes: tetraploid, pentaploid, hexaploid and decaploid. Remarkable is the high number, 85 out of 186 accessions, of the pentaploid cytotype. Although it is largely sterile, bulbs seem to be a very effective way for vegetative multiplication, just as found for *G. spathacea* (Pfeiffer et al. 2012). *Gagea lutea* is only found in an inferred hexaploid form. The nonaploidy reported for *G. spathacea* would suggests a low basic genome size. This is corroborated by the fact that *G. spathacea* belongs to a section different from the others. Flow cytometry could provide the correct identification in most cases. It is a taxonomic and diagnostic tool that is applicable even in the case of dormant bulbs or sterile plants, and therefore has applications for conservation monitoring. Future research of the Dutch gageas could focus on combining chromosome counts and flow cytometry of the same samples, especially in the case of *G. villosa*. The fertility of the pentaploid *G. pratensis* needs further investigation. Sequencing of the forma *glauca* of *G. lutea* could reveal if it is a separate species or not.
